# Widely Targeted Metabolomics Analysis Reveals the Differences of Nonvolatile Compounds in Oolong Tea in Different Production Areas

**DOI:** 10.3390/foods11071057

**Published:** 2022-04-06

**Authors:** Zhihui Wang, Shuang Gan, Weijiang Sun, Zhidan Chen

**Affiliations:** 1 College of Horticulture, Fujian Agriculture and Forestry University, Fuzhou 350002, China; wzh1246900265@163.com (Z.W.); ganshuang1995@163.com (S.G.); 2 Anxi College of Tea Science, Fujian Agriculture and Forestry University, Fuzhou 350002, China

**Keywords:** Oolong tea, widely targeted metabolomics, nonvolatile compounds, producing areas, taste

## Abstract

The flavor differences in Oolong tea from different producing areas are caused by its complex differential compounds. In this study, representative samples of Oolong tea from four countries were collected, and their differential nonvolatile compounds were analyzed by a combination of widely targeted metabolomics, chemometrics, and quantitative taste evaluation. A total of 801 nonvolatile compounds were detected, which could be divided into 16 categories. We found that the difference in these compounds’ content among Oolong teas from three producing areas in China was the largest. There were 370 differential compounds related to the producing areas of Oolong tea, which were mainly distributed in 67 Kyoto Encyclopedia of Genes and Genomes (KEGG) metabolic pathways. In total, 81 differential nonvolatile compounds made important contributions to the taste differences in Oolong tea from different producing areas, among which the number of flavonoids was the largest. Finally, the characteristic compounds of Oolong tea in six producing areas were screened. This study comprehensively identifies the nonvolatile compounds of Oolong tea in different producing areas for the first time, which provides a basis for the analysis of flavor characteristics, quality directional control, and the identification and protection of geographical landmark agricultural products of Oolong tea from different producing areas.

## 1. Introduction

Tea (*Camellia sinensis* (L.) *O.* *kuntze*) is one of the most traditional nonalcoholic drinks in the world, with unique aromas, tastes, and health benefits [1]. According to the difference in the tea processing technology used, it can be divided into six types—namely, green tea, black tea, yellow tea, Oolong tea, dark tea, and white tea [2]. Oolong tea is a semi-fermented tea that is mainly produced in the Fujian, Guangdong, and Taiwan provinces of China [3]. Oolong tea was first produced in the early Song Dynasty (960–1279) but became popular in the Ming Dynasty (1368–1644). The traditional manufacturing process of Oolong tea includes withering, shaking, de-enzyming, rolling, and drying [3]. The quality characteristics of traditional Oolong tea are green leaves with red edges, golden infusion, and an elegant fruity and floral, thick, and refreshing taste [4]. Oolong tea is favored by consumers for its unique flavor and quality [4]. Today, Oolong tea is produced in many countries and regions, including Japan, Myanmar, Sri Lanka, South Korea, etc. [5]. The flavor formation of Oolong tea is affected by the variety, producing areas, processing technology, season, and picking method [6]. For example, different Oolong tea cultivars have different “cultivar fragrances” [3]. Multiple stresses (low/high temperature, machinery, and air humidity) in processing technology can cause Oolong tea to accumulate different metabolites [7]. Therefore, there are great differences in the quality characteristics of Oolong tea from different producing areas. The tea plant is a treasure-house of many natural bioactive metabolites, and these metabolites are not only the key contributing factors to tea color, aroma, and taste quality but are also the main providers of tea health care functions [8]. There is no systematic report on the flavor differences and nonvolatile compounds of Oolong tea in different countries and regions, which is not conducive to people’s deep and extensive understanding of the characteristics of Oolong tea in different producing areas. Studies of flavor formation of Oolong tea from the level of small molecular nonvolatile compounds play an important role in clarifying the formation mechanism of the flavor differences in Oolong tea from different producing areas.

Plant metabolomics is a qualitative and quantitative analysis of all metabolites in plants [9]. It is a technology that combines a holistic and comprehensive analysis and chemical informatics analysis methods to determine the target differential metabolites so as to clarify the metabolic process and change mechanism in organisms [9,10]. In recent years, this technology has been gradually applied in the field of tea research to reveal the growth and development of the tea plant, the formation mechanism of tea flavor, the evaluation of tea grade and quality, and the traceability and discrimination of teas [11,12]. Targeted metabolomics and non-targeted metabolomics are often used to carry out metabolomics research, but these two methods have some defects [13]. Targeted metabolomics technology has high data accuracy and reliability but limited coverage of metabolites. On the other hand, non-targeted metabolomics technology is characterized by high coverage of compounds but has a low accuracy [13]. In contrast, widely targeted metabolomics combine the advantages of non-targeted and targeted metabolite detection technology to achieve high throughput, high sensitivity, and wide coverage [13]. At present, this technology has achieved good results in the flavor analysis of horticultural crops and foods [13,14]. Wang et al. [14] used widely targeted metabolomics to reveal the changes in metabolites and the nutritional quality of Lycium barbarum fruits in different production areas. Zou et al. [15] used widely targeted metabolomics to determine that the taste differences among different loquat cultivars could be explained by the changes in the composition and content of carbohydrates, organic acids, amino acids, and phenolic compounds. Wang et al. [16] revealed the dynamic changes of nonvolatile and volatile metabolites during green tea processing by widely targeted metabolomics. Wu et al. [17] used widely targeted metabolomics to study the nonvolatile metabolites in the processing of Oolong tea and found that carbohydrates, amino acids, and flavonoids may contribute to the comprehensive flavor of Oolong tea.

In view of this, this study aimed to analyze the differential nonvolatile compounds of Oolong tea from different producing areas by combining widely targeted metabolomics and chemometrics to clarify the metabolic pathways involved in these differential compounds and determine the characteristic compounds of Oolong tea from six producing areas. At the same time, combined with the quantitative taste evaluation, the compounds that had an important impact on the taste differences of Oolong tea from different producing areas were screened. These results will provide a theoretical basis for analyzing the flavor differences and production quality control of Oolong tea in different countries and regions and provide a reference for the origin traceability of Oolong tea in different geographical regions.

## 2. Materials and Methods

### 2.1. Experimental Materials

In order to collect representative samples of Oolong tea produced in different countries, we learned from the “World Tea Production Data Information Table” [18] and other relevant literature [5] that, in addition to China, Japan, Myanmar, and Sri Lanka also produce a large amount of Oolong tea. Therefore, 36 samples of Oolong tea products were collected from four different countries, including 8 from Guangdong, China, 8 from Northern Fujian, China, 7 from Southern Fujian, China, 5 from Japan, 3 from Sri Lanka, and 5 from Myanmar. These Oolong tea samples included different grades and different cultivars. Sample details are shown in Table S1. After collection, the samples were stored in the refrigerator at −80 °C for standby. First, the tastes of all samples were preliminarily evaluated by referring to GB/T 23776–2018 [19]. We excluded samples with similar taste characteristics from the same producing areas. Finally, twelve representative Oolong tea samples from various producing areas were then selected for taste quantitative evaluation and widely targeted metabolomics analysis, including 3 from China (1 from Guangdong (GD), 1 from Northern Fujian (MB), and 1 from Southern Fujian (MN)), 3 from Japan (J1, J2, and J3), 3 from Sri Lanka (S1, S2, and S3), and 3 from Myanmar (M1, M2, and M3) (Table S1).

### 2.2. Experimental Method

#### 2.2.1. Nonvolatile Compounds Extraction

The sample to be tested was vacuum freeze-dried and ground into powder (30 Hz, 1.5 min). Then, 100 mg of the ground sample was weighed and placed in a 2 mL centrifuge tube, and 1 mL of 70% methanol was added and mixed evenly; this mixture was put in the refrigerator at 4 °C for extraction for 12 h. During this period, it was vortex oscillated three times to improve the extraction rate. After that, it was centrifuged (Centrifuge 5810 R, Rotor S-4-104, Eppendorf, Hamburg, Germany) at 10,000 g-forces for 10 min, and the supernatant was absorbed with a 0.22 μm microporous membrane filtration, which was stored in the injection bottle to be tested by liquid chromatography–electrospray ionization–tandem mass spectrometry (LC-ESI-MS/MS). Three biological replicates were taken for each sample.

#### 2.2.2. Quality Control Samples Processing

The quality control samples (QC) were prepared by equal mixing of 6 groups of Oolong tea extracts from different producing areas. They were treated and tested by the same method as the analytical samples and repeated 6 times. In the process of instrument testing, one QC sample was inserted into every 10 test and analysis samples to monitor the repeatability of the whole analysis process.

#### 2.2.3. LC-ESI-MS/MS Analysis of Nonvolatile Compounds

Nonvolatile compounds were identified using an LC-ESI-MS/MS system (HPLC, Shim-pack UFLC SHIMADZU CBM30A, Kyoto, Japan; MS, Applied Biosystems 6500 Q TRAP, Kyoto, Japan). For the HPLC conditions, the chromatographic column used was Waters ACQUITY UPLC HSS T3 C18 (2.1 mm × 100 mm, 1.8 µm, Waters Company, Milford, MA, USA). For the mobile phase, phase A was 0.04% acetic acid, and phase B was acetonitrile solution containing 0.04% acetic acid. The elution gradient of phase B was 0.00–11.00 min, 5–95%, maintained at 95%, 1.00 min; 11.00–12.10 min, 95–5%, and balanced at 5% until 15.00 min. The flow rate was 0.4 mL/min, the column temperature was 40 °C, and the injection volume was 2 μL.

For the mass spectrometry parameters, the electrospray ionization (ESI) temperature was 500 °C, the mass spectrum voltage was 5500 V, the curtain gas (CUR) was 25 psi, and the parameter of the collision-activated dissociation (CAD) was set to high. In the triple quadrupole, each ion pair was scanned and detected according to the optimized declustering potential (DP) and collision energy (CE).

#### 2.2.4. Quantitative Evaluation of Taste Attributes of Oolong Tea from Different Producing Areas

According to the requirements of GB/T 16291.1-2012 [20], five evaluators were selected from the tea science teacher team of Fujian Agriculture and Forestry University to form a sensory evaluation team. The five evaluators had participated in various sensory evaluation experiments such as black tea, white tea, and Oolong tea and had more than 10 years of experience in sensory tea evaluation. The taste quantitative evaluation method used to assess the twelve representative Oolong tea samples was based on GB/T 23776-2018 (the covered-bowl method) with modifications [19]. The evaluation team used a 0–5 strength scale standard to evaluate the intensities of bitterness, astringency, umami, “sweet aftertaste”, and “heavy and thick” of the samples, namely 0 = none and 5 = very. Briefly, 5.0 g of the sample was weighed, and placed in a 110 mL covered bowl, filled with boiling water, and capped. Tea was brewed three times. After the first was brewed for 2 min, the tea infusion was drained; the second was brewed for 3 min, and the third was brewed for 5 min. The taste intensity was evaluated immediately after the tea infusion was drained each time. The result was the average of the three evaluations. Every sample was randomly evaluated three times.

### 2.3. Statistical Analysis

The nonvolatile compounds were analyzed qualitatively by comparing the ion fragment mode, retention time, and m/z value combined with the self-compiled database (MetWare, Wuhan, China) [21] and the public databases and quantified by using the multiple reaction monitoring (MRM) mode of triple quadrupole mass spectrometry. The nonvolatile compounds data were processed and analyzed by Analyst 1.6.3 software. One-way analysis of variance (ANOVA) with least significant difference (LSD) was performed using SPSS19.0. Principal component analysis (PCA), orthonormal partial least-squares discriminant analysis (OPLS-DA), and orthogonal partial least squares analysis (O2PLS) were performed using SIMCA 14.0. Heat maps were generated using Hiplot software [22]. The correlation data network diagram of O2PLS was generated using Cytoscape 3.9.1 software.

## 3. Results and Discussion

### 3.1. Profile Analysis of Taste Characteristics of Oolong Tea from Different Producing Areas

**Figure 1:**
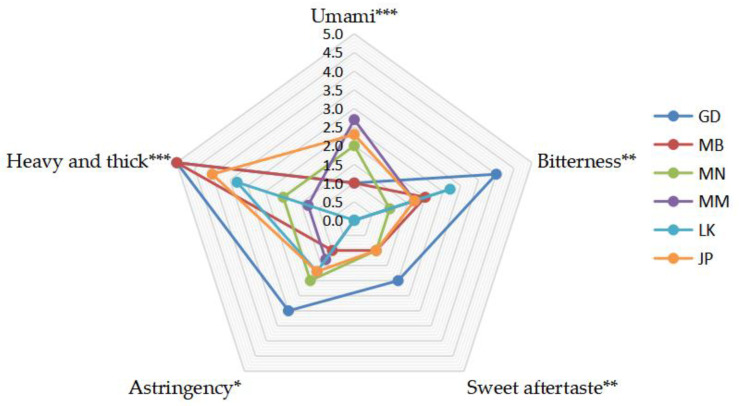
Radar map of the taste characteristics of Oolong tea from different producing areas. The taste quantitative evaluation data shown in the radar map are the mean values of Oolong tea samples in the same producing area. GD, Guangdong, China; MB, Northern Fujian, China; MN, Southern Fujian, China; MM, Myanmar; LK, Sri Lanka; JP, Japan. * *p* < 0.05, ** *p* < 0.01, *** *p* < 0.001.

The mean values of the evaluation results of the taste intensity of Oolong tea from different producing areas are shown in Figure 1. There were statistically significant differences in the intensity of five taste attributes of Oolong tea in six producing areas (Figure 1). The taste characteristics of Guangdong and Northern Fujian’s Oolong tea had the strongest intensity of “heavy and thick”. The difference between them was that Guangdong’s Oolong tea had a strong intensity of a sweet aftertaste, bitterness, and astringency, while Northern Fujian’s Oolong tea had a weak intensity of bitterness and astringency. The intensities of bitterness and “heavy and thick” of the Oolong tea from Southern Fujian were weaker than those in Northern Fujian and Guangdong, but the umami was stronger than both. Southern Fujian’s Oolong tea is mainly Tieguanyin, Northern Fujian’s Oolong tea is mainly Wuyi rock tea, and Guangdong’s Oolong tea is mainly Fenghuang Dancong tea [3]. Previous studies on the quality characteristics of Oolong tea from these three producing areas found that each had its own characteristics, but there was no quantitative evaluation of taste [6,23]. In this study, the quantitative taste evaluation was carried out to further clarify the taste differences of Oolong tea in the three production areas. Myanmar and Japan’s Oolong tea had a similar intensity of umami, bitterness, sweet aftertaste, and astringency, but the difference was that Japan’s Oolong tea had a stronger “heavy and thick” taste. Sri Lanka’s Oolong tea had the weakest intensities of umami and sweet aftertaste, and the intensities of “heavy and thick” and an astringent taste were relatively strong.

### 3.2. Identification of Nonvolatile Compounds of Oolong Tea from Different Producing Areas

**Figure 2:**
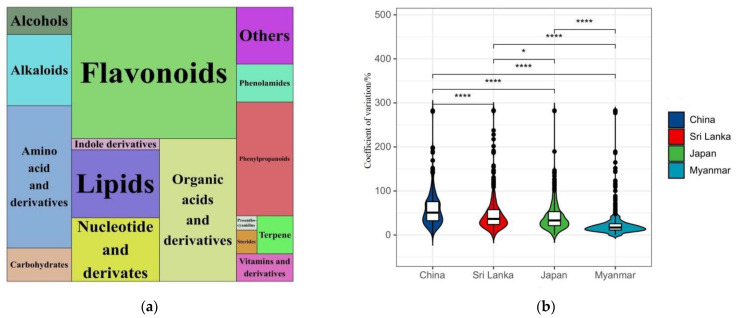
Statistical analysis of nonvolatile components of Oolong tea from different producing areas. (**a**) Tree view of nonvolatile components classification. The larger the area and text of the component category in the figure is, the larger the number and proportion of the component category is. Each color represents a class of compounds. (**b**) Violin chart of coefficient of variation of components of Oolong tea in four countries. * *p* < 0.05, **** *p* < 0.001.

Widely targeted metabolomics analysis based on LC–ESI–MS/MS was conducted on the nonvolatile compounds of Oolong tea from four countries. Through database comparison, a total of 801 compounds was identified (Table S2). These compounds could be divided into 16 categories (Figure 2a). They were ranked according to the number and proportion of nonvolatile compounds included in each category, which were flavonoids (221, 27.6%), organic acids and their derivatives (112, 14.0%), amino acids and their derivatives (94, 11.7%), phenylpropanoids (66, 8.2%), lipids (61, 7.6%), nucleotides and their derivates (57, 7.1%), alkaloids (47, 5.9%), others (33, 4.1%), phenolamides (22, 2.7%), saccharides (22, 2.7%), alcohols (18, 2.2%), vitamins and their derivatives (16, 2.0%), terpenes (14, 1.7%), indole derivatives (10, 1.2%), sterides (5, 0.6%), and proanthocyanidins (3, 0.4%).

The overall difference in the content of Oolong tea nonvolatile compounds between the four countries was large, and the average coefficient of variation reached 44.1% (Figure 2b). The order of the mean value of the coefficient of variation was China (58.2%) > Sri Lanka (48.6%) > Japan (42.9%) > Myanmar (26.6%) (Figure 2b). Among them, China’s Oolong tea samples (Northern Fujian, Southern Fujian, and Guangdong) showed the largest difference in nonvolatile components, followed by Sri Lanka and Japan, and the smallest in Myanmar.

### 3.3. Analysis of Differential Nonvolatile Compounds and Metabolic Pathways in Oolong Tea from Different Producing Areas

**Figure 3:**
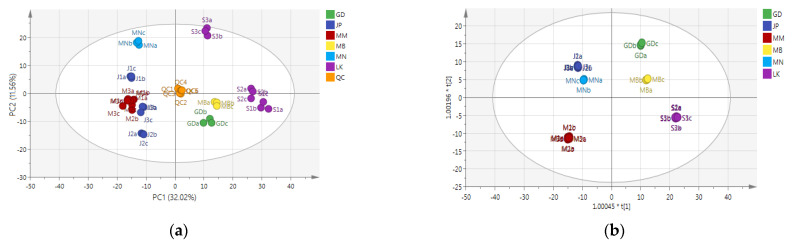

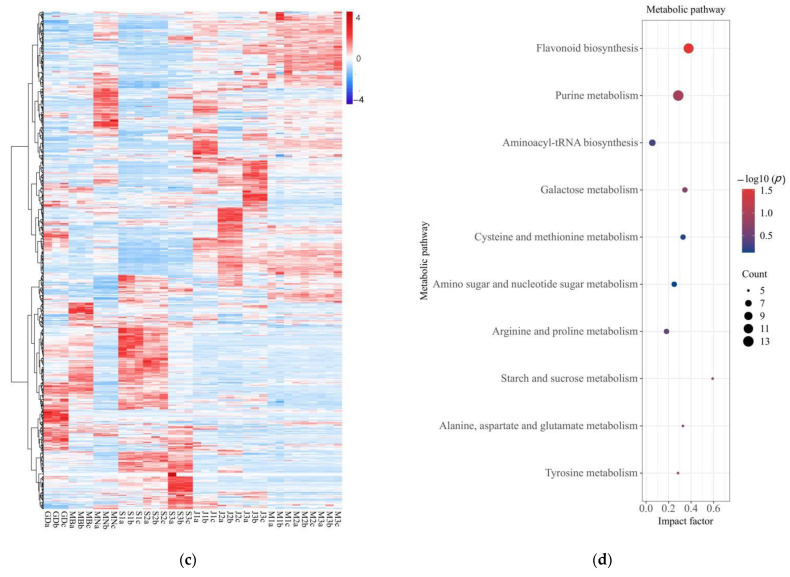
Differential nonvolatile compound in Oolong tea from different producing areas. (**a**) PCA score chart, R^2^X = 0.848, Q^2^ = 0.711. (**b**) OPLS-DA score chart, R^2^X = 0.900, R^2^Y = 0.998, Q^2^ = 0.987. (**c**) Differential nonvolatile compound heat map. Red indicates high content and blue indicates low content. (**d**) Ten metabolic pathways with the highest enrichment. GD, Guangdong, China; MB, Northern Fujian, China; MN, Southern Fujian, China; MM, Myanmar; LK, Sri Lanka; JP, Japan; QC, quality control sample. a, b, and c represent three biological repetitions of metabolome samples.

PCA can form new characteristic variables by a linear combination of variables according to a certain weight. Because there are no external human factors, the PCA model can reflect the original state of the data [24]. PCA was performed with all detected nonvolatile compounds as variables, and the results are shown in Figure 3a. Six QC samples were gathered in the middle of the PCA score plot, and the three repetitions of each sample could be effectively aggregated, indicating good repeatability and reliable data. In the PCA score chart, the distribution of Oolong tea samples from the three producing areas in China was the most dispersed, followed by Sri Lanka and Japan, and Myanmar was clustered together. These results indicated that the difference in nonvolatile compounds in the Oolong tea samples from the three producing areas in China was the largest, followed by Sri Lanka and Japan, while the difference between Myanmar’s Oolong tea was the smallest. This conclusion was exactly the same as that of the coefficient of variation (Figure 2b). The samples of Oolong tea from Japan and Myanmar were relatively clustered on the PCA score plot, indicating that the content of nonvolatile compounds of Oolong tea from the two countries was relatively similar.

Compared with PCA, OPLS-DA provides a supervised classification, which can effectively distinguish samples and extract the information of differential variables [25]. In order to question the differential nonvolatile compounds and distinguish Oolong tea from the six producing areas, OPLS-DA modeling analysis was carried out on all the detected compounds, and it was discovered that Oolong tea from the six producing areas was effectively distinguished in the model (Figure 3b). Cross-validation analysis manifested that the model had a high degree of interpretation (R^2^X = 0.900, R^2^Y = 0.998, Q^2^ = 0.987). The substitution test graph showed that the model was not overfitted (all blue Q^2^ points from left to right were lower than the original green Q^2^ points at the far right, and the intersection of the regression line of Q^2^ points at the ordinate was less than 0) (Figure S1). Differential compounds were screened according to the principle of VIP > 1.00 and *p* < 0.05 [26]. A total of 370 differential nonvolatile compounds were identified, including 14 categories, namely, flavonoids (103), organic acids and their derivatives (54), amino acids and their derivatives (38), phenylpropanoids (31), nucleotides and their derivates (31), alkaloids (25), others (20), lipids (19), saccharides (16), vitamins and their derivatives (9), phenolamides (7), alcohols (7), terpenes (6), and indole derivatives (4) (Table S3). These 370 differential nonvolatile compounds were closely related to the producing areas of Oolong tea.

The total heat map of the differential compounds of the sample is shown in Figure 3c. The difference in nonvolatile compounds among samples from three producing areas of China was the largest (the distribution of red and blue regions was different). S3 in Sri Lanka had different nonvolatile compounds than S1 and S2 (the distribution of red and blue regions of S3 was different from that of S1 and S2). The contents of differential compounds in Japan and Myanmar were partially similar. These results were the same as the conclusion of the PCA (Figure 3a) and the coefficient of variation (Figure 2b).

Since there was no complete metabolomics database for tea plants, Arabidopsis thaliana was taken as the corresponding species for KEGG metabolic pathway analysis. The screened differential compounds were mainly enriched in 67 metabolic pathways (Table S4), of which the 10 metabolic pathways with the highest enrichment were respectively: flavonoid biosynthesis, purine metabolism, aminoacyl-tRNA biosynthesis, galactose metabolism, cysteine and methionine metabolism, amino sugar and nucleotide sugar metabolism, arginine, and proline metabolism, starch, and sucrose metabolism, alanine, aspartate and glutamate metabolism, and tyrosine metabolism (Figure 3d). Flavonoid biosynthesis mainly included the metabolism of flavonoids, flavonoid glycosides, catechins, and anthocyanins [27]. The products of purine metabolism also participated in the biosynthesis of alkaloids [28]. Aminoacyl-tRNA biosynthesis, cysteine and methionine metabolism, amino sugar and nucleotide sugar metabolism, arginine and proline metabolism, alanine, aspartate, and glutamate metabolism, and tyrosine metabolism belonged to the synthesis and degradation of amino acids [29]. Galactose metabolism and starch and sucrose metabolism were mainly the synthesis and transformation of saccharides [30]. Therefore, the difference in the content of nonvolatile compounds in Oolong tea from different producing areas mainly stemmed from the metabolism of flavonoids, amino acids, saccharides, and alkaloids in Oolong tea. These four substances are also the main contributing components of the Oolong tea flavor [3].

### 3.4. Differential Nonvolatile Compounds Analysis and Characteristic Nonvolatile Compounds Identification of Oolong Tea from Different Producing Areas

In order to screen out the characteristic compounds of Oolong tea in various producing areas, 370 differential nonvolatile compounds (VIP > 1.00, *p* < 0.05) were analyzed using a heat map. The screening criteria for characteristic compounds were based on the variation trend of variables in the heat map. Variables with a high content (red) in all samples from the same producing area and low content (blue and white) in all samples from other producing areas were regarded as characteristic compounds [31].

#### 3.4.1. Flavonoids

**Figure 4:**
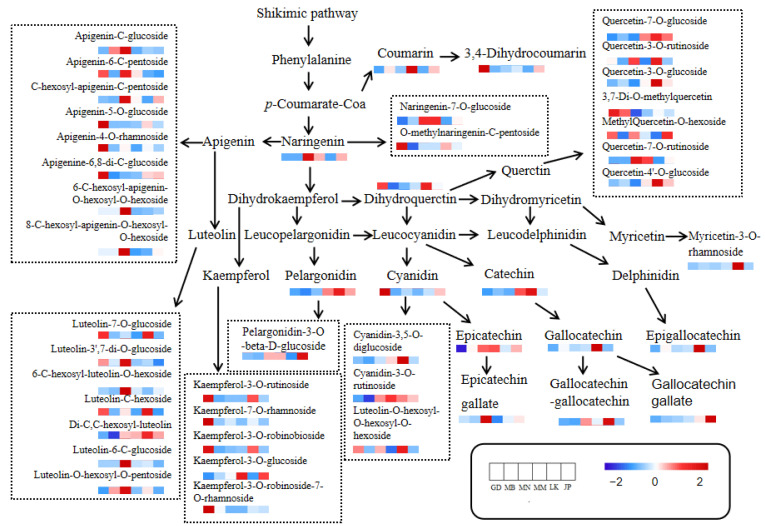
Distribution of differential flavonoid substances in the flavonoid metabolism pathway of Oolong tea from different producing areas. GD, Guangdong, China; MB, Northern Fujian, China; MN, Southern Fujian, China; MM, Myanmar; LK, Sri Lanka; JP, Japan. Red indicates high content and blue indicates low content. The metabolic pathway of flavonoids comes from the literature [26].

Flavonoids are the main polyphenols in tea, including flavonols, flavonoids, flavanols, flavanones, anthocyanins, and isoflavones, among which flavanols (mainly catechins) and flavonols (such as quercetin and its glycosides) are the main subclasses in tea [32]. Heat map analysis was conducted on the differential flavonoids (Figure S2), and their differential metabolic pathways are shown in Figure 4. The content of flavonoids of Oolong tea from different producing areas was significantly different, mainly flavonols and catechins. Guangdong’s Oolong tea had a high content of flavonoid glycosides, such as kaempferol glycosides, apigenin glycosides, some luteolin glycosides, and quercetin glycosides. The contents of catechins, anthocyanins, and some flavonoid glycosides in Sri Lanka’s Oolong tea were high, such as catechin, (+)-gallocatechin, epigallocatechin, geranium pigment glycosides, myricetin, and some quercetin glycosides. The contents of naringin, some apigenin glycosides, some luteolin glycosides, and some catechins in Southern Fujian’s Oolong tea were higher. The content of flavonoids in Japan’s Oolong tea and Myanmar’s Oolong tea was low. The content of flavonoids in Northern Fujian’s Oolong tea was the lowest. Catechins and flavonols are the main contributors to the bitterness and astringency of tea infusion; for example, ester catechins, quercetin glycosides, and myricetin glycosides would make the mouth feel bitter and astringent [33]. Scharbert et al. [34] claimed that flavanol glycosides were important substances in the astringency of tea infusion and could enhance the bitterness of caffeine. Chen et al. [35] reported that catechins and their oxidation products were important substances in the bitter and astringent taste of Oolong tea. In sensory evaluation (Figure 1), Guangdong’s Oolong tea had the strongest bitterness and astringency, followed by Sri Lanka, and was weaker when from Japan, Myanmar, and Northern Fujian. The intensities of bitterness and astringency of the tea infusions were consistent with the content trend of flavonoids in the samples, suggesting that flavonoids, especially flavonols, catechins, and anthocyanins, might be the main contribution to the difference in bitterness and astringency of Oolong tea. According to the screening method of characteristic compounds, the selected characteristic flavonoids were kaempferol-7-O-rhamnoside in Guangdong’s Oolong tea, myricetin-3-O-rhamnoside, isoliquiritigenin, epigallocatechin, and (+)-gallocatechin in Sri Lanka’s Oolong tea, and (+)-gallocatechin and deoxyrhapontin in Myanmar’s Oolong tea.

#### 3.4.2. Amino Acids and Their Derivatives

Amino acids, mainly theanine, glutamic acid, and aspartic acid, are the main contributors to the umami of tea infusion and can weaken the bitter and astringent taste [36]. Differential amino acids and derivatives were analyzed by a heat map (Figure S3). Japan and Myanmar’s Oolong tea had a higher amino acid content, while the amino acid content of Northern Fujian and Sri Lanka’s Oolong tea was lower than others. In the sensory evaluation, the umami intensity of Japan and Myanmar’s Oolong tea was stronger, while that of Sri Lanka and Northern Fujian was weaker. The trends in umami intensity and the content of amino acids and their derivatives were relatively uniform, indicating that amino acids and their derivatives might have an important impact on the umami differences of Oolong tea from different producing areas. The roasting process can lead to theanine degradation and produce pyrazines with a roasted aroma [37]. The roasting aroma of Oolong tea in Northern Fujian and Sri Lanka’s Oolong tea was produced by the roasting process, which might be an important reason for the low amino acid content of both. Based on the different profiles in the heat map, the selected characteristic amino acids and their derivatives were lysine butyrate and N-acetylglycine in Guangdong’s Oolong tea, N-acetyl-L-phenylalanine in Sri Lanka’s Oolong tea, S-5’-adenosyl-L-methionine and DL-homocysteine in Japan’s Oolong tea, and H-homoarg-OH and N-acetyl-L-leucine in Myanmar’s Oolong tea.

#### 3.4.3. Saccharides

The saccharides in tea include monosaccharides, disaccharides, oligosaccharides, polysaccharides, and traces of other saccharides. The content of soluble saccharides in Oolong tea is high [38]. Saccharides mainly affect the sweetness and viscosity of the tea infusion and inhibit the bitterness, as well as optimize the taste of tea, and also affect the aroma, such as the roasted smell produced by the Maillard reaction [36]. The heat map analysis of differential saccharides (Figure S4) showed that the content of saccharides in Japan and Southern Fujian’s Oolong tea was higher. Japan’s Oolong tea had higher fructose, glucose, and mannose, and Southern Fujian’s Oolong tea contained higher galactose, maltose, pine trisaccharide, and pentose. The saccharide content in Myanmar and Guangdong’s Oolong tea was moderate. The contents of glucose, fructose, and mannose in Northern Fujian and Sri Lanka’s Oolong tea were low, which might be related to their long-term roasting, which caused the saccharides to participate in the Maillard reaction and convert into aroma substances. The selected characteristic saccharides were ribulose-5-phosphate in Guangdong’s Oolong tea, N-acetyl-D-glucosamine in Northern Fujian’s Oolong tea, and maltotetraose and panose in Southern Fujian’s Oolong tea.

#### 3.4.4. Alkaloids

Alkaloids and their salts are mostly bitter, some are extremely bitter and spicy, and some have the burning feeling of stimulating the lips and tongue and can generate synergistic effects with other bitter substances [39]. Caffeine, theobromine, and theophylline are the main alkaloids in tea [40]. However, these three important alkaloids did not belong to the differential compounds of Oolong tea in different producing areas. Heat map analysis results of differential alkaloids (Figure S5) indicated that the alkaloid content of Oolong tea from Guangdong and Northern Fujian was higher, while that of Myanmar, Japan, and Southern Fujian was lower. In sensory evaluation, the bitterness of Guangdong’s Oolong tea was superior, and that of Northern Fujian’s Oolong tea was slightly weak. It may be due to the interaction between flavonoids and alkaloids in Guangdong’s Oolong tea leading to enhanced bitterness. The selected characteristic alkaloids were betaine, 4-phenyldiazenylbenzene-1,3-diamine, and hydrochloride in Guangdong’s Oolong tea, indole-3-carboxylic, and tryptophol in Northern Fujian’s Oolong tea, peimine, 2-hydroxypyridine, and l-dencichin in Southern Fujian’s Oolong tea, and 4-pyridoxic acid in Japan’s Oolong tea.

#### 3.4.5. Organic Acids and Their Derivatives

Organic acids are important intermediate metabolites in the tricarboxylic acid cycle and shikimic acid pathway and play an important role in the flavor of tea [41]. The content of organic acids is the highest in black tea, followed by Oolong tea [36]. In this study, it was found that organic acids and their derivatives had great differences in Oolong tea from different producing areas, and the identified differential nonvolatile compounds were second only to flavonoids. The heat map analysis of differential organic acids and their derivatives showed that the content of organic acids and their derivatives in Myanmar and Sri Lanka’s Oolong tea was relatively high, while those in Japan and China were relatively low (Figure S6). There were great differences in organic acids and their derivatives in Oolong tea from the three producing areas in China. The contents of sebacate, L-(+)-Tartaric acid, 10-Formyl-THF, D-tartaric acid, and methyl gallate in Guangdong were higher, while the contents of p-Hydroxyphenyl acetic acid, mandelic acid, 5-O-p-Coumaroyl shikimic acid, and 3-O-p-Coumaroyl shikimic acid in Northern Fujian were higher, and the contents of 2-Hydroxybutanoic acid, α-Hydroxyisobutyric acid, anisic acid-O-feruloyl hexoside, and 2-Methylsuccinic acid in Southern Fujian were higher. At present, there are few studies on organic acids in Oolong tea. In the process of black tea processing, it was found that the effect of withering and rolling on organic acid accumulation was more important than fermentation [41]. The process of withering and rolling is also used in the processing of Oolong tea, and the roasting process can increase the content of gallic acid [42]. Therefore, the differences in organic acids and their derivatives in Oolong tea from different producing areas may have a greater relationship with the production process. The selected characteristic organic acids and their derivatives were 2-Methoxybenzoic acids in Northern Fujian’s Oolong tea and neochlorogenic acid in Myanmar’s Oolong tea.

#### 3.4.6. Phenylpropanoids, Nucleotides, Lipids and Others

Nucleotide and its derivatives have umami, which can enhance the mellow and sweet taste [43]. Phenylpropanoid participates in the shikimic acid metabolic pathway and is the synthetic precursor of many taste and aroma substances [44]. Lipid is an important component of the biofilm system, one of the six human nutrients, as well as the synthetic precursors of volatile compounds and some bitter substances [45]. Up to now, there has been little research on the contribution of these substances to the tea taste, which needs to be further discussed. A heat map was used to analyze the differential nucleotides (Figure S7), phenylpropanoids (Figure S8), lipids (Figure S9), and others (Figure S10) in the samples. The results manifested that the nucleotide content of Oolong tea was higher in Guangdong and Sri Lanka’s Oolong tea and lower in Southern Fujian’s Oolong tea. Myanmar’s Oolong tea had higher phenylpropanoid content. The lipid content of Sri Lanka, Myanmar, and Japan’s Oolong tea was relatively high, while that of China’s Oolong tea was relatively low; among them, the lipid content of Southern Fujian’s Oolong tea was extremely low. The selected characteristic compounds were 6-hydroxymethylhernirin in Guangdong’s Oolong tea, syringic acid, 7-hydroxy-5-methoxycoumarin, and ribopline in Myanmar’s Oolong tea, β- nicotinamide mononucleotide in Japan’s Oolong tea, α-linolenic acid in Sri Lanka’s Oolong tea, and indole-5-carboxylic acid and indole-3-carboxaldehyde in Northern Fujian’s Oolong tea.

**Figure 5:**
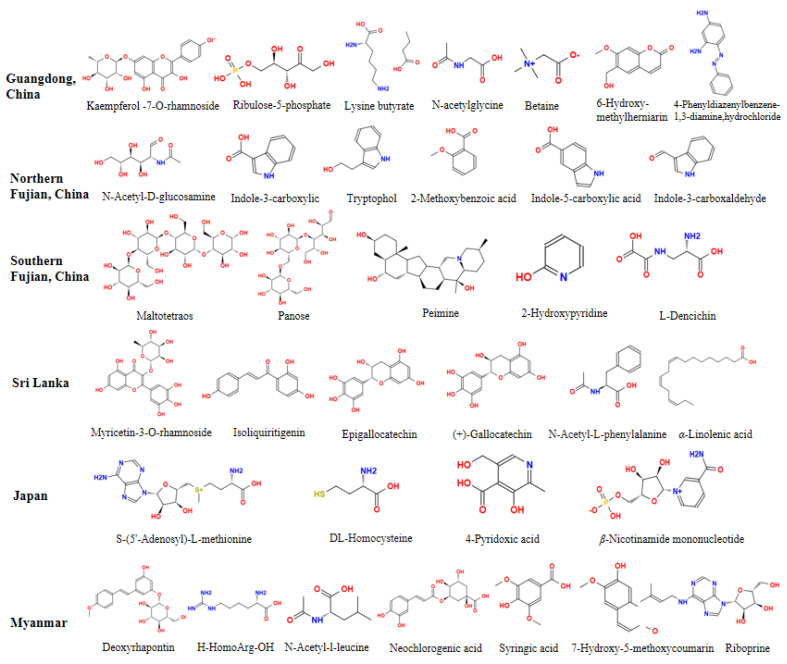
Characteristic nonvolatile compounds screened from Oolong tea from six producing areas.

To sum up, 35 characteristic compounds were screened from Oolong tea from different producing areas (Figure 5), including seven in Guangdong, six in Northern Fujian, five in Southern Fujian, six in Sri Lanka, four in Japan, and seven in Myanmar. These selected characteristic compounds were the compounds specifically accumulated by Oolong tea in a certain producing area, which could be applied to the traceability identification of Oolong tea from this producing area and other producing areas. The reasons for the specific accumulation of characteristic compounds of Oolong tea from these producing areas need to be further studied.

### 3.5. Relationship between Differential Nonvolatile Compounds and Taste Characteristics of Oolong Tea from Different Producing Areas

The unique taste quality of tea is reflected in various chemical components, and its taste difference comes from the difference in the composition and content of these chemical components [46]. In order to identify the components that made an important contribution to the taste differences in Oolong tea from different producing areas, an O2PLS model was established with 370 differential compounds (X variable) and five taste sub-attributes (Y variable) as variables (Figures S11 and S12). Under the criteria of the VIP value > 1.0, *p* < 0.05, and correlation coefficient >0.7 [47], 81 nonvolatile compounds were identified as compounds with an important contribution to taste sub-attributes, including 19 flavonoids, 14 organic acids and their derivatives, 11 phenylpropanoids, 10 amino acids and their derivatives, 8 nucleoside acids and their derivatives, 6 saccharides, 6 others, 5 lipids, and 2 alkaloids. The difference in the content of these 81 nonvolatile compounds in Oolong tea from different producing areas may be the main reason for the difference in taste.

**Figure 6:**
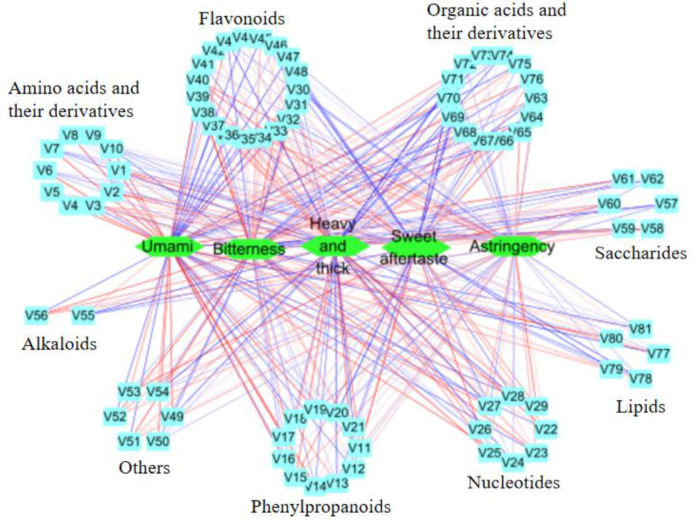
Correlation network between differential nonvolatile compounds and taste attributes in Oolong tea from different producing areas. V1–V81 indicate 81 differential compounds (compound names are shown in Table S5). The red line indicates a positive correlation, and the blue line indicates a negative correlation, and the darker the color, the stronger the correlation.

The correlation coefficient and correlation network diagram are shown in Table S5 and Figure 6, respectively. Among the 81 compounds, the number of flavonoids was the largest, mainly including flavonols and their glycosides, catechins, and anthocyanins and their glycosides. Most of these substances were positively correlated with “heavy and thick”, bitterness, and astringency (more red lines) and negatively correlated with umami (more blue lines) (Figure 6). This result confirmed the above speculation that flavonols, catechins, and anthocyanins may be important contributors to the difference in the bitterness and astringency of Oolong tea. The processing technology of Oolong tea has a great impact on flavonoids [17]. Fermentation and roasting can promote the decrease in catechins, and roasting can reduce flavonoid glycosides and procyanidins so as to weaken the bitterness and astringency of Oolong tea [42]. At the same time, the tea cultivar is also the main reason for the difference in flavonoid content in Oolong tea [48]. Most amino acids and derivatives were positively correlated with umami (more red lines) (Figure 6). The differences in amino acids and their derivatives mainly come from the cultivar and processing technology of tea [29]. Many reports have shown that flavonoids, amino acids, saccharides, and alkaloids have important effects on the taste of Oolong tea [3,23,42], but the effects of organic acids and their derivatives, phenylpropanoids, nucleotides and their derivatives, and lipids on the taste differences in Oolong tea have not been systematically studied, especially organic acids and their derivatives. This study showed that the effect of organic acids and their derivatives on the taste differences in Oolong tea in different production areas was second only to flavonoids, more than the number of amino acids and their derivatives, and its different mechanisms need to be further explored.

## 4. Conclusions

In this paper, for the first time, we used widely targeted metabolomics, chemometrics, and taste quantitative evaluation to reveal the difference in nonvolatile compounds and the mechanism of the taste differences in Oolong tea from different producing areas. This work provided comprehensive information on the composition and content of nonvolatile compounds in Oolong tea from different producing areas. In total, 801 nonvolatile compounds were detected using widely targeted metabolomics, including 370 differential compounds. These differential compounds were mainly distributed across 67 KEGG metabolic pathways. The metabolic pathways with a high enrichment level were closely related to the metabolism of flavonoids, amino acids, saccharides, and alkaloids. There were statistically significant differences in the intensity of the five taste attributes of Oolong tea from six producing areas. In total, 81 nonvolatile compounds had an important contribution to the taste difference, among which the number of flavonoids was the largest, followed by organic acids and their derivatives, phenylpropanoids, and amino acids and their derivatives. Finally, the characteristic compounds of Oolong tea from six producing areas were screened.

## Data Availability

The data presented in this study are available within the manuscript and the Supplementary Materials (Tables S1–S5, Figures S1–S12).

## Supplementary Materials

The following supporting information can be downloaded at: https://www.mdpi.com/article/10.3390/foods11071057/s1, Figure S1: OPLS-DA permutations plot; Figure S2: Heat map analysis of differential flavonoids; Figure S3: Heat map analysis of differential amino acid and their derivatives; Figure S4: Heat map analysis of differential saccharides; Figure S5: Heat map analysis of differential alkaloids; Figure S6: Heat map analysis of differential organic acids and their derivatives; Figure S7: Heat map analysis of differential nucleotides and their derivates; Figure S8: Heat map analysis of differential phenylpropanoids; Figure S9: Heat map analysis of differential lipids; Figure S10: Heat map analysis of differential others; Figure S11: O2PLS score chart; Figure S12: O2PLS permutations plot; Table S1: Information of Oolong tea samples in different producing areas; Table S2: Detection results of nonvolatile compounds in Oolong tea from different producing areas; Table S3: Differential nonvolatile compounds and their VIP values in Oolong tea from different producing areas; Table S4: KEGG metabolic pathway enrichment information; Table S5: Correlation between differential nonvolatile compounds and taste attributes.
