# Supplementary material for: Widely Targeted Metabolomics Analysis Reveals the Differences of Nonvolatile Compounds in Oolong Tea in Different Production Areas
Source: Foods. 2022 Apr 6;11(7):1057. doi: 10.3390/foods11071057 (PMC8998066; doi:10.3390/foods11071057)
Supplement: Supplementary file 1 [file foods-11-01057-s001.zip › Support information.pdf]

Table S1. Information of Oolong tea samples in different producing areas

Sample number (Metabolome sample)	Sample source	Commodity information
GD1	Guangdong, China	Super Shuixian
GD2		First class Shuixian
GD3 (GDa, GDb, GDc)		Super Dancong (Fenghuangdancong)
GD4		First class Dancong (Fenghuangdancong)
GD5		Second class Dancong (Fenghuangdancong)
GD6		Heimeiren
GD7		Guihuaxiang
GD8		Dashu Tea
MN1	South Fujian, China	Dangui
MN2		Dayewulong
MN3		Jinguanyin
MN4 (MNa, MNb, MNc)		Tieguanyin
MN5		Huangdan
MN6		Maoxie
MN7		Benshan
MB1	North Fujian, China	Jinmudan
MB2 (MBa, MBb, MBc)		Dahongpao (Wuyi Rock Tea)
MB3		Super Rougui
MB4		First class Rougui
MB5		Second class Rougui
MB6		Super Shuixian
MB7		First class Shuixian
MB8		Second class Shuixian
JP1 (J1a, J1b, J1c)	Japan	Japanese market (Chunxun)
JP2 (J2a, J2b, J2c)		Japanese market (Flower)
JP3 (J3a, J3b, J3c)		Japanese market (Fangkou)
JP4		Japanese market (Shuang)
JP5		Japanese market (Granular oolong tea)
MM1 (M1a, M1b, M1c)	Myanmar	Myanmar market (High mountain Oolong tea)
MM2 (M2a, M2b, M2c)		Myanmar market
MM3 (M3a, M3b, M3c)		Myanmar market
MM4		Myanmar market (High mountain Oolong tea)
MM5		Myanmar market
LK1 (S1a, S1b, S1c)	Sri Lanka	Sri Lanka (I. S.B. experts)
LK2 (S2a, S2b, S2c)		Sri Lanka (Ceylon oolong tea)
LK3 (S3a, S3b, S3c)		Sri Lanka market

Note: GD, Guangdong, China; MB, Northern Fujian, China; MN, Southern Fujian, China; MM, Myanmar; LK, Sri Lanka; JP, Japan. GD3 (GDa, GDb, GDc) represents metabolome samples, and a, b, and c represent three biological repetitions. Others represent the same.

Table S4. KEGG metabolic pathway enrichment information

Name	Hits	Impact	p
Purine metabolism	13	0.28602	0.10886
Flavonoid biosynthesis	12	0.37781	0.029514
Aminoacyl-tRNA biosynthesis	7	0.05556	0.50411
Galactose metabolism	6	0.34489	0.18319
Cysteine and methionine metabolism	6	0.32858	0.67231
Amino sugar and nucleotide sugar metabolism	6	0.25028	0.75105
Arginine and proline metabolism	6	0.18157	0.36537
Starch and sucrose metabolism	5	0.59154	0.20132
Alanine, aspartate and glutamate metabolism	5	0.32734	0.20132
Tyrosine metabolism	5	0.28379	0.06786
Ascorbate and aldarate metabolism	5	0.23881	0.10482
Tryptophan metabolism	5	0.22222	0.3791
Phenylpropanoid biosynthesis	5	0.10174	0.81706
Isoquinoline alkaloid biosynthesis	4	1	0.0049915
Pentose and glucuronate interconversions	4	0.09375	0.18958
Citrate cycle (TCA cycle)	4	0.23269	0.32429
Valine, leucine and isoleucine biosynthesis	4	0.05721	0.39461
Inositol phosphate metabolism	4	0.10251	0.59197
Glyoxylate and dicarboxylate metabolism	4	0.03371	0.62111
Glycine, serine and threonine metabolism	4	0.10708	0.72401
Valine, leucine and isoleucine degradation	4	0.00991	0.80475
Flavone and flavonol biosynthesis	3	0	0.16463
Anthocyanin biosynthesis	3	0.33333	0.20354
Nicotinate and nicotinamide metabolism	3	0.0606	0.28614
Arginine biosynthesis	3	0.10097	0.49336
Lysine degradation	3	0.5	0.49336
Pentose phosphate pathway	3	0.37301	0.5316
Propanoate metabolism	3	0.10591	0.56819
Zeatin biosynthesis	3	0.0271	0.60297
Carbon fixation in photosynthetic organisms	3	0.17556	0.60297
Glutathione metabolism	3	0.35113	0.74808
Glycerophospholipid metabolism	3	0.09397	0.92062
Glucosinolate biosynthesis	3	0	0.99757
Betalain biosynthesis	2	1	0.056282
Vitamin B6 metabolism	2	0.03205	0.48639
Sulfur metabolism	2	0.0663	0.66056
Butanoate metabolism	2	0.13636	0.72828

beta-Alanine metabolism	2	0.14683	0.75764
Fructose and mannose metabolism	2	0.1351	0.80824
Glycerolipid metabolism	2	0.05957	0.82985
Pyruvate metabolism	2	0	0.84925
Thiamine metabolism	2	0	0.84925
Phenylalanine, tyrosine and tryptophan biosynthesis	2	0.11011	0.84925
Pantothenate and CoA biosynthesis	2	0	0.86662
Glycolysis / Gluconeogenesis	2	0.10295	0.90832
Ubiquinone and other terpenoid-quinone biosynthesis	2	0.00097	0.98122
Pyrimidine metabolism	2	0.02076	0.98122
Linoleic acid metabolism	1	0	0.46422
Indole alkaloid biosynthesis	1	0.5	0.46422
Biosynthesis of secondary metabolites - unclassified	1	1	0.54174
Biosynthesis of secondary metabolites - other antibiotics	1	0	0.6081
One carbon pool by folate	1	0	0.71347
Stilbenoid, diarylheptanoid and gingerol biosynthesis	1	0.13235	0.71347
Lysine biosynthesis	1	0	0.75504
Caffeine metabolism	1	0	0.7906
Riboflavin metabolism	1	0.11852	0.82102
Phenylalanine metabolism	1	0.23529	0.82102
Nitrogen metabolism	1	0	0.84704
Histidine metabolism	1	0	0.90459
Sphingolipid metabolism	1	0.03365	0.93039
Biotin metabolism	1	0.07692	0.94055
Biosynthesis of unsaturated fatty acids	1	0	0.96841
Phosphatidylinositol signaling system	1	0.03285	0.98325
alpha-Linolenic acid metabolism	1	0.10665	0.98781
Cyanoamino acid metabolism	1	0	0.9896
Terpenoid backbone biosynthesis	1	0	0.99113
Fatty acid biosynthesis	1	0	0.99986

Table S5. Correlation between differential nonvolatile compounds and taste attributes

Number	Non-volatile compounds	Umami	Bitterness	Sweet aftertaste	Astringency	Heavy and thick
V1	N6-Acetyl-L-lysine	-0.094	0.469	0.729	0.433	0.701
V2	L-Theanine	-0.028	0.045	0.532	0.208	0.712
V3	L-Tyramine	-0.628	0.730	0.250	0.415	0.483
V4	L-(-)-Tyrosine	0.715	-0.234	0.233	0.015	0.104
V5	5-Hydroxy-L-tryptophan	0.737	-0.344	0.190	0.062	-0.013
V6	S-(5'-Adenosy)-L-homocysteine	0.793	-0.320	-0.092	-0.245	-0.463
V7	L-Glutamine	0.827	-0.254	-0.376	-0.278	-0.567
V8	N,N-Dimethylglycine	0.805	-0.164	0.017	-0.096	-0.224

V9	Allysine(6-Oxo DL-Norleucine)	0.786	-0.503	0.109	-0.019	-0.269
V10	γ-aminobutyric acid	0.822	-0.288	-0.211	-0.314	-0.388
V11	Ferulic acid	-0.424	0.533	0.611	0.383	0.713
V12	4-Methylumbelliferone	-0.584	0.204	0.666	0.303	0.758
V13	Syringic acid	0.487	-0.243	-0.435	-0.273	-0.773
V14	3-(4-Hydroxyphenyl)propionic acid	0.809	-0.451	0.201	0.148	-0.578
V15	3-Hydroxy-4-methoxycinnamic acid	-0.430	0.540	0.603	0.382	0.700
V16	Trans-cinnamaldehyde	0.867	-0.403	0.005	-0.159	-0.445
V17	Syringin	0.803	-0.322	0.323	0.166	-0.057
V18	6,7-Dihydroxycoumarin-6-glucoside	-0.702	0.192	-0.024	-0.063	0.546
V19	Xanthotoxol	0.805	-0.327	-0.311	-0.419	-0.456
V20	Brevifolincarboxylic acid	-0.222	0.114	-0.885	-0.411	-0.431
V21	Isofraxidin	0.131	0.055	-0.488	-0.175	-0.730
V22	2'-Deoxyinosine-5'-monophosphate	-0.565	0.243	0.440	0.197	0.733
V23	Purine	-0.662	0.733	0.223	0.396	0.492
V24	5'-Deoxy-5'-(methylthio)adenosine	0.710	-0.428	-0.246	-0.304	-0.698
V25	7-Methylxanthine	-0.001	0.767	0.081	0.210	0.511
V26	β-Pseudouridine	-0.134	-0.189	0.743	0.418	0.578
V27	UDP-α-D-glucose	0.627	-0.637	-0.457	-0.435	-0.771
V28	Riboprine	0.424	-0.187	-0.599	-0.306	-0.770
V29	Inosine 5'-monophosphate	-0.515	0.505	0.533	0.704	0.519
V30	Peonidin O-hexoside	0.531	-0.704	-0.359	-0.558	-0.455
V31	Cyanidin	-0.047	0.701	0.255	0.216	0.474
V32	Peonidin 3-O-glucoside chloride	0.581	-0.720	-0.292	-0.471	-0.414
V33	Tricin	-0.538	0.796	0.283	0.394	0.586
V34	Luteolin 7-O-glucoside	-0.755	0.394	-0.014	0.160	0.369
V35	Chrysoeriol O-glucuronic acid	-0.719	0.245	-0.417	-0.109	0.125
V36	Quercetin 3-O-rutinoside	-0.723	0.131	-0.068	0.078	0.239
V37	Kaempferol 3-O-rutinoside	-0.713	0.616	0.139	0.378	0.388
V38	Kaempferol 7-O-rhamnoside	-0.323	0.713	0.437	0.525	0.440
V39	3,7-Di-O-methylquercetin	-0.375	0.821	0.170	0.084	0.649
V40	Kaempferol-3-O-robinoside-7-O-rhamnoside	-0.128	0.293	0.744	0.719	0.529
V41	Kaempferol 3-O-glucoside	0.812	-0.539	-0.139	-0.281	-0.545
V42	Hesperetin 7-O-neohesperidoside	-0.722	0.127	-0.038	0.089	0.268
V43	Naringenin 7-O-glucoside	0.591	-0.703	-0.230	-0.381	-0.717

V44	Hesperetin 7-rutinoside	-0.710	0.135	-0.044	0.098	0.254
V45	Isoliquiritigenin	-0.799	0.470	-0.377	-0.049	0.272
V46	Catechin	-0.354	0.216	-0.867	-0.365	-0.406
V47	Epigallocatechin	-0.774	0.254	-0.480	-0.119	0.009
V48	(+)-Gallocatechin	-0.784	0.249	-0.497	-0.135	-0.003
V49	3-Indoleacetonitrile	0.739	-0.526	-0.091	-0.120	-0.735
V50	Oryzalexin E	0.726	-0.568	-0.014	-0.319	-0.259
V51	4-Methyl-5-thiazoleethanol	-0.707	0.786	0.154	0.432	0.415
V52	Hydroxyphenethylamine	-0.667	0.762	0.217	0.416	0.486
V53	Glucarate O-Phosphoric acid	0.542	-0.345	0.743	0.311	0.275
V54	Mangiferin	0.486	-0.524	-0.299	-0.300	-0.788
V55	N-Methyltryptamine	0.298	-0.173	-0.657	-0.379	-0.702
V56	Dendrobine	-0.621	0.711	0.268	0.383	0.521
V57	D(+)-Melezitose	0.016	-0.786	-0.179	-0.442	-0.469
V58	D(-)-Threose	-0.732	0.782	0.111	0.438	0.396
V59	D(+)-Glucose	0.800	-0.459	0.214	0.105	-0.148
V60	D-Fructose 6-phosphate	0.327	-0.338	0.739	0.332	0.228
V61	D-(+)-Mannose	0.779	-0.528	0.315	0.099	-0.080
V62	Panose	0.033	-0.791	-0.055	-0.379	-0.373
V63	Syringaldehyde O-glucoside	0.475	-0.496	-0.199	-0.300	-0.717
V64	p-Coumaroyl quinic acid O-glucuronic acid	-0.691	0.812	0.118	0.476	0.422
V65	Methyl gallate	-0.042	0.571	0.703	0.663	0.554
V66	Chlorogenic acid	-0.749	0.561	-0.363	0.052	0.240
V67	4-Hydroxybenzoic acid	-0.080	-0.070	-0.764	-0.550	-0.373
V68	Gallic acid	-0.655	0.716	0.231	0.340	0.560
V69	Chlorogenic acid methyl ester	0.024	0.101	-0.873	-0.361	-0.664
V70	Succinic acid	0.729	-0.576	0.516	0.059	-0.019
V71	Quinic acid	-0.021	-0.188	-0.797	-0.468	-0.733
V72	Citric acid	-0.011	-0.273	-0.616	-0.317	-0.771
V73	Fumaric acid	0.585	-0.821	-0.003	-0.203	-0.651
V74	α-Hydroxyisobutyric acid	0.250	-0.538	-0.135	0.047	-0.720
V75	1-O-Caffeoyl quinic acid	-0.738	0.523	-0.383	0.015	0.235
V76	Aminomalonic acid	0.691	-0.713	0.459	-0.015	-0.140
V77	LysoPC 18:1	-0.764	0.601	-0.307	0.156	0.192
V78	LysoPC 18:3	0.313	0.182	-0.773	-0.291	-0.572
V79	LysoPC 18:2	0.212	0.296	-0.771	-0.252	-0.430
V80	4-oxo-9Z,11Z,13E,15E-octadecatetraenoic acid	0.722	-0.303	0.245	0.076	0.121
V81	Delta-Tridecalactone	0.200	-0.719	-0.171	-0.479	-0.186

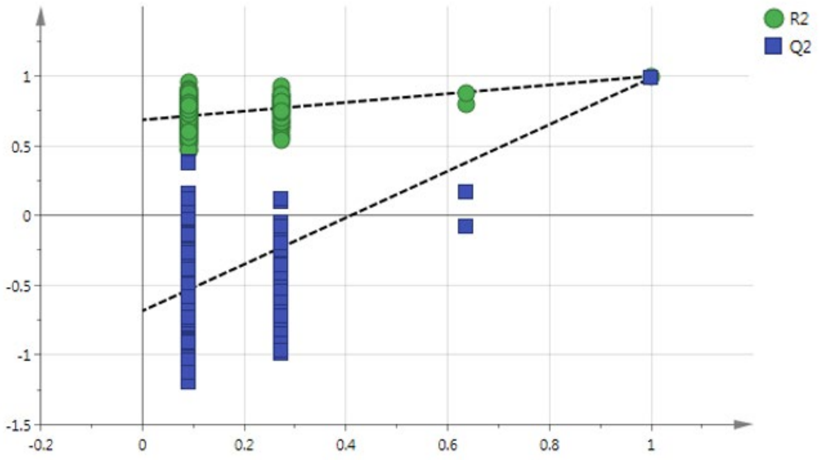

Figure S1. OPLS-DA permutations plot

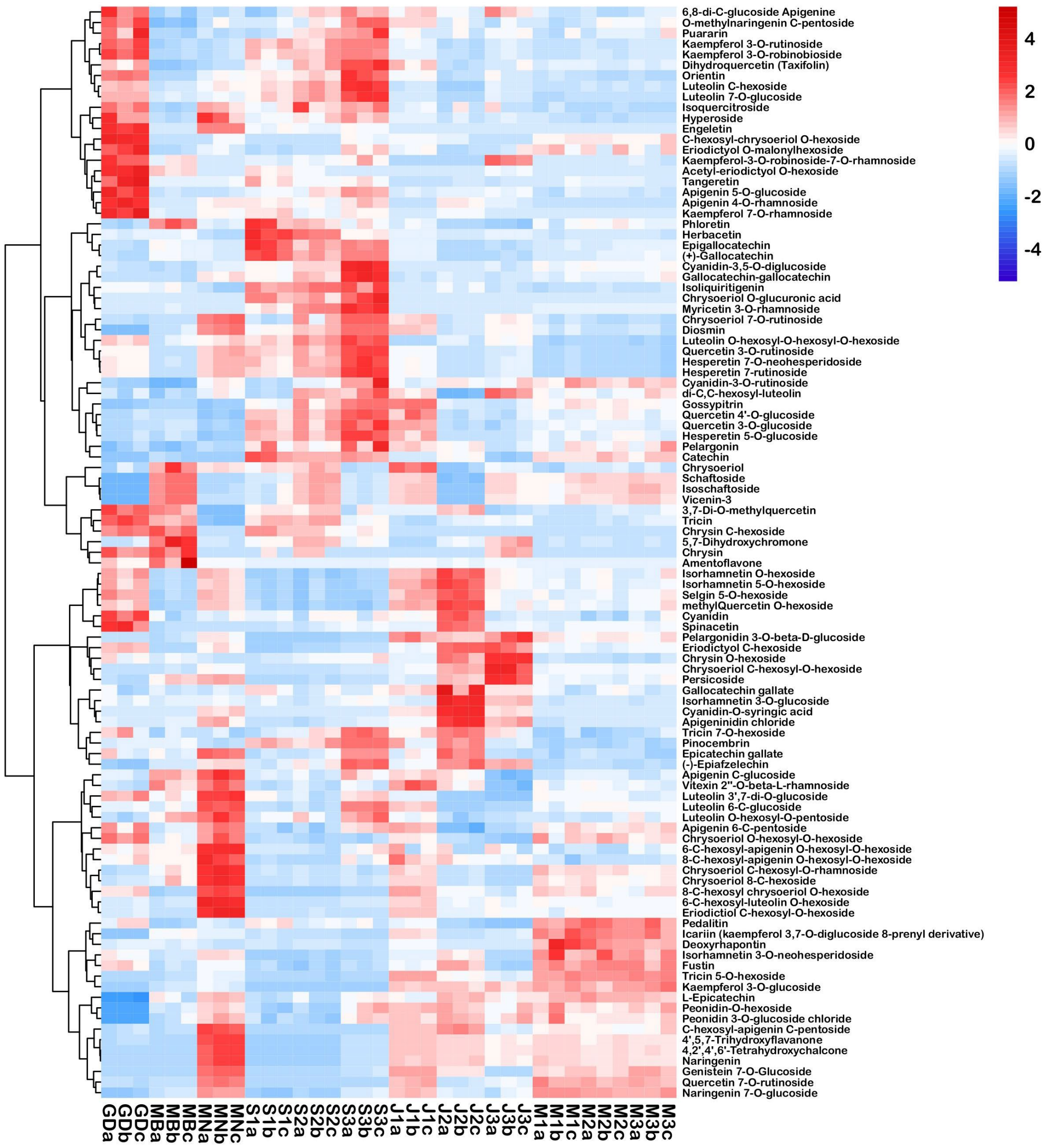

Figure S2. Heat map analysis of differential flavonoids. GD, Guangdong, China; MB, Northern Fujian, China; MN, Southern Fujian, China; MM, Myanmar; LK, Sri Lanka; JP, Japan. Red indicates high content and green indicates low content. $P < 0.05$.

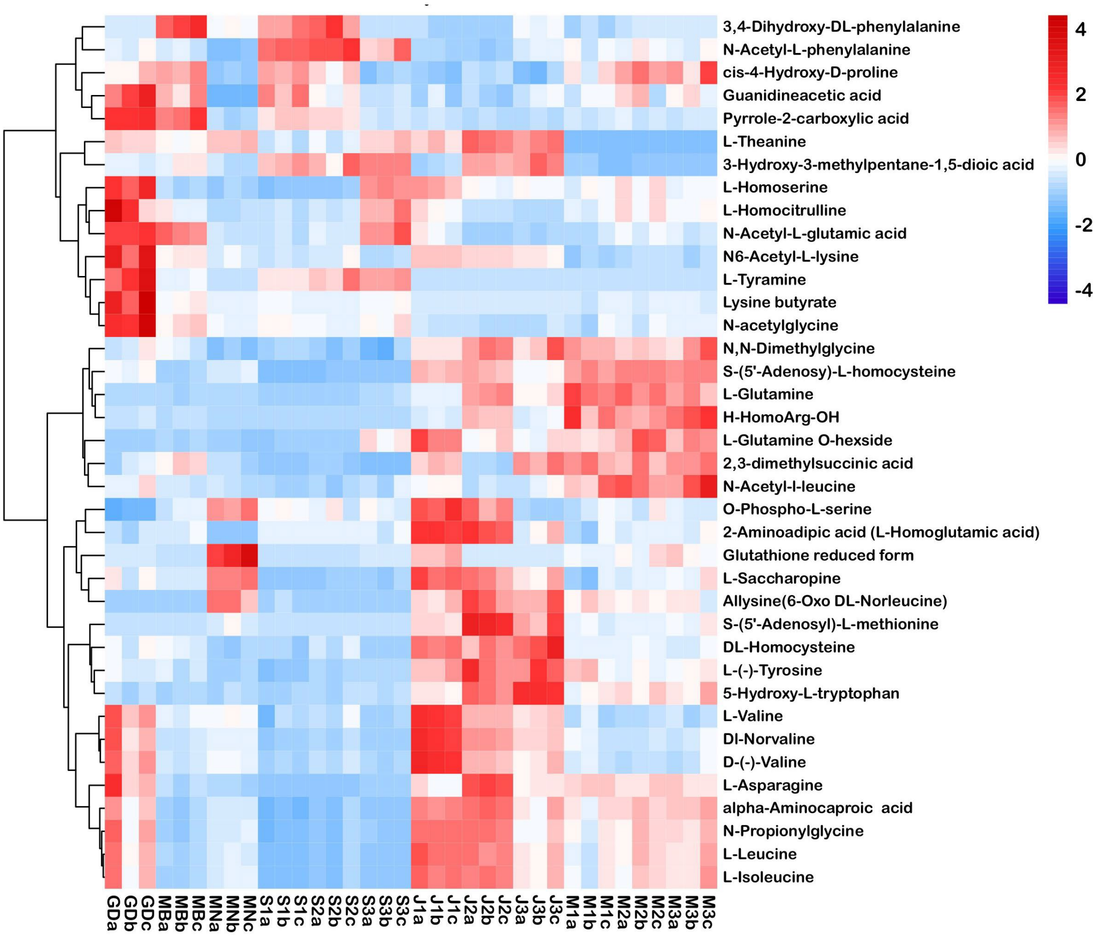

Figure S3. Heat map analysis of differential amino acid and their derivatives. GD, Guangdong, China; MB, Northern Fujian, China; MN, Southern Fujian, China; MM, Myanmar; LK, Sri Lanka; JP, Japan. Red indicates high content and green indicates low content. $P < 0.05$.

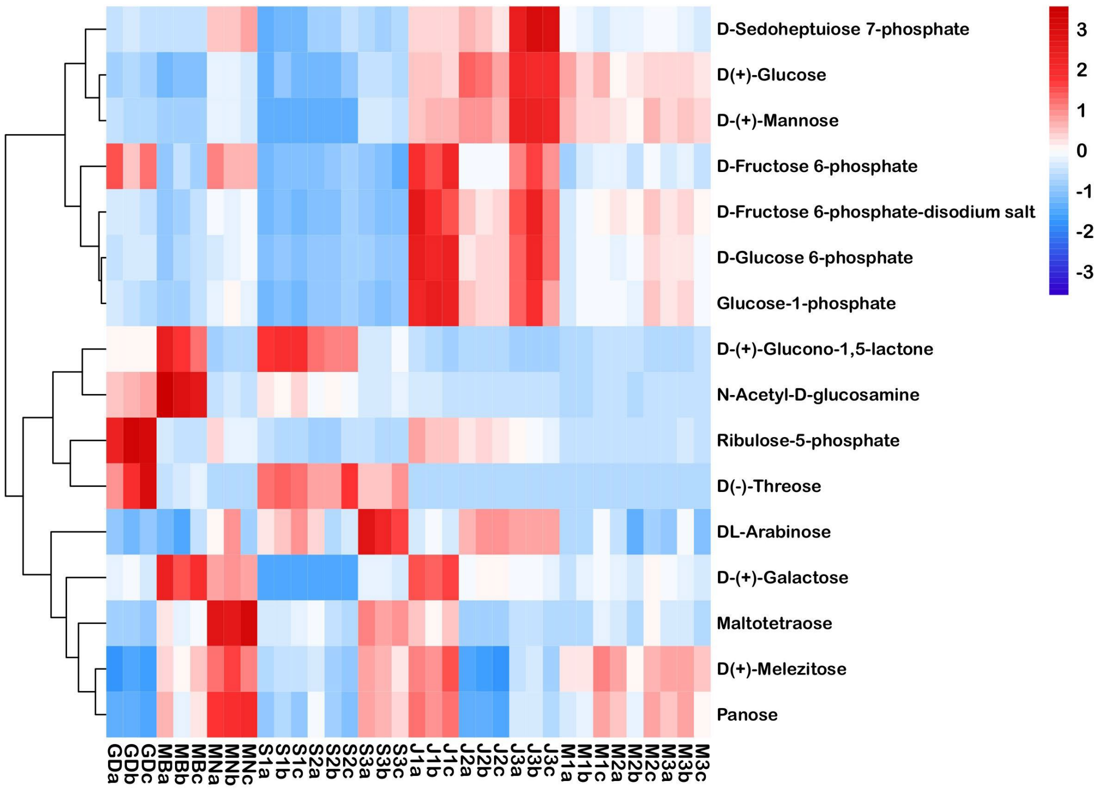

Figure S4. Heat map analysis of differential saccharides. GD, Guangdong, China; MB, Northern Fujian, China; MN, Southern Fujian, China; MM, Myanmar; LK, Sri Lanka; JP, Japan. Red indicates high content and green indicates low content. $P < 0.05$.

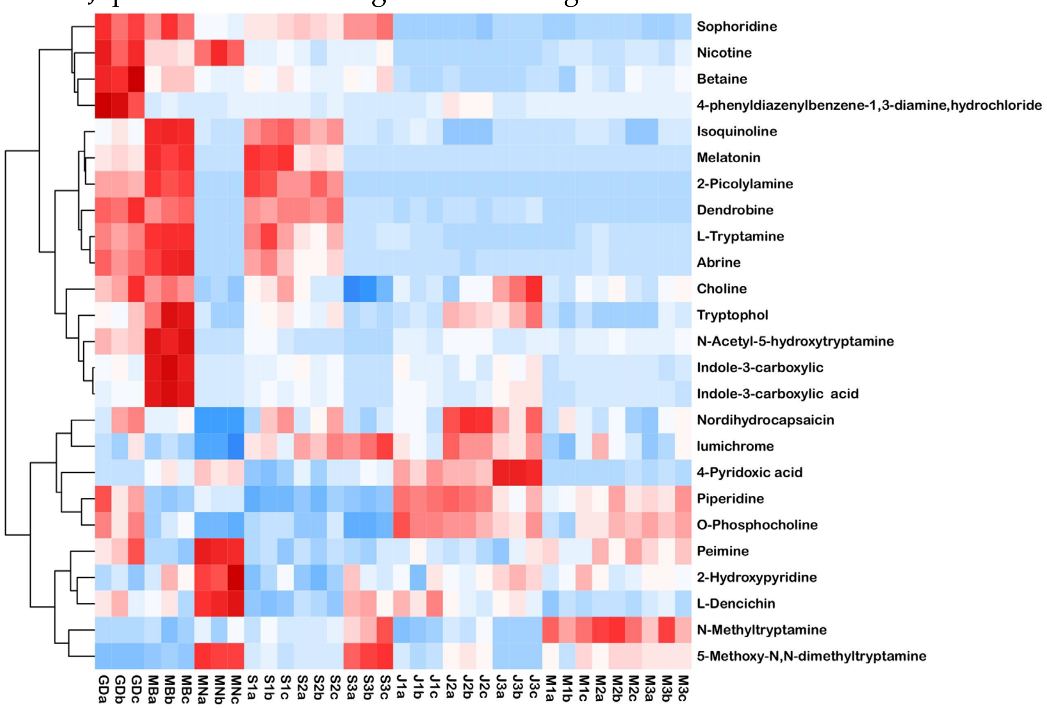

Figure S5. Heat map analysis of differential alkaloids. GD, Guangdong, China; MB, Northern Fujian, China; MN, Southern Fujian, China; MM, Myanmar; LK, Sri Lanka; JP, Japan. Red indicates high content and green indicates low content. $P < 0.05$.

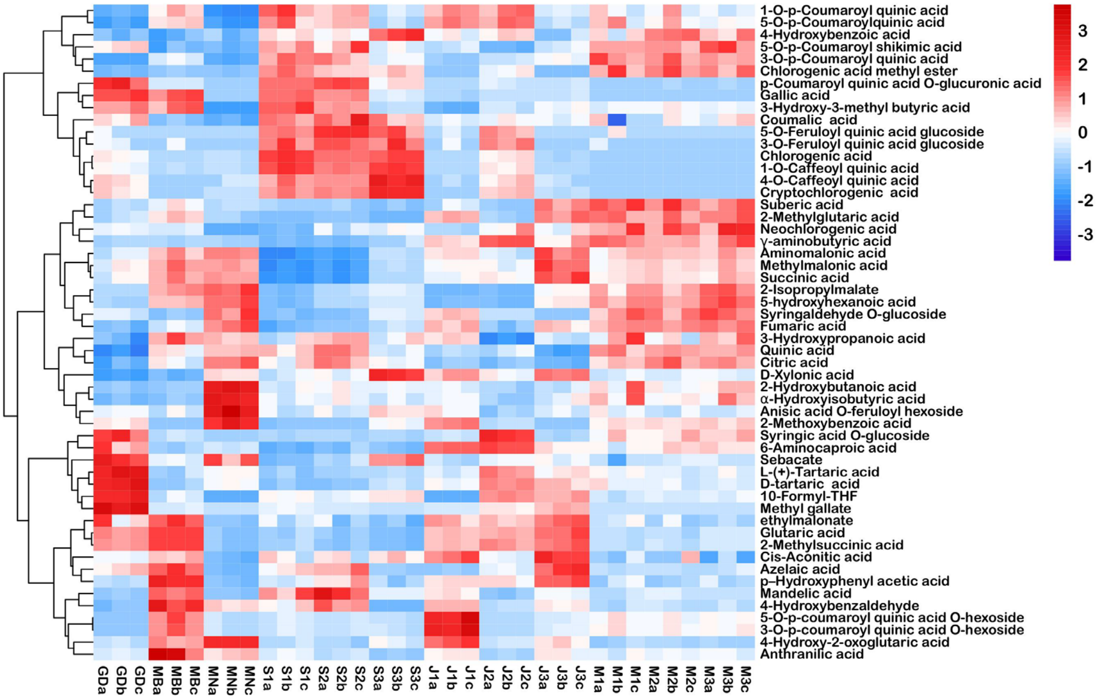

Figure S6. Heat map analysis of differential organic acids and their derivatives. GD, Guangdong, China; MB, Northern Fujian, China; MN, Southern Fujian, China; MM, Myanmar; LK, Sri Lanka; JP, Japan. Red indicates high content and green indicates low content. $P < 0.05$.

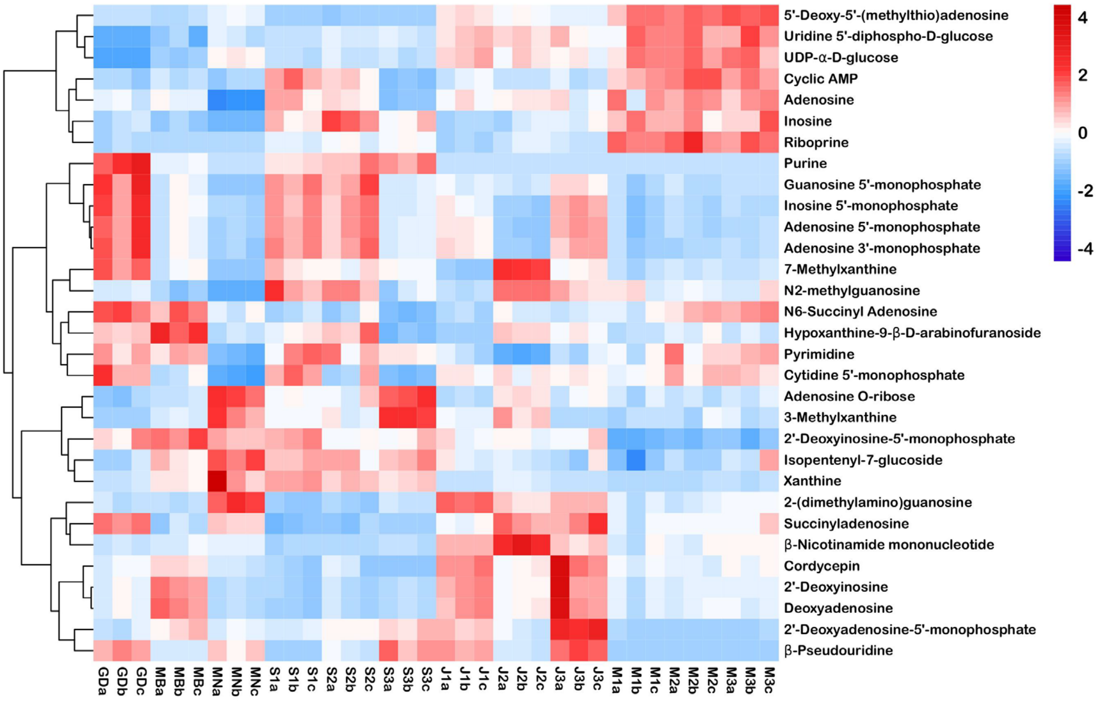

Figure S7. Heat map analysis of differential nucleotides and their derivates. GD, Guangdong, China; MB, Northern Fujian, China; MN, Southern Fujian, China; MM, Myanmar; LK, Sri Lanka; JP, Japan. Red indicates high content and green indicates low content. $P < 0.05$.

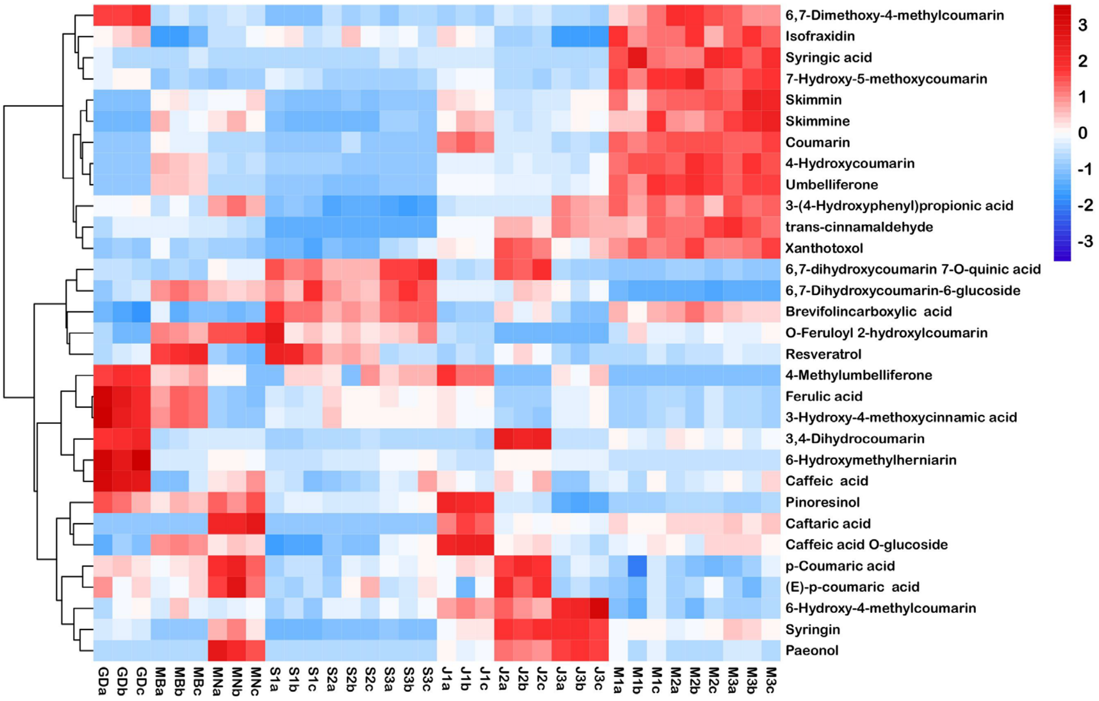

Figure S8. Heat map analysis of differential phenylpropanoids. GD, Guangdong, China; MB, Northern Fujian, China; MN, Southern Fujian, China; MM, Myanmar; LK, Sri Lanka; JP, Japan. Red indicates high content and green indicates low content. $P < 0.05$.

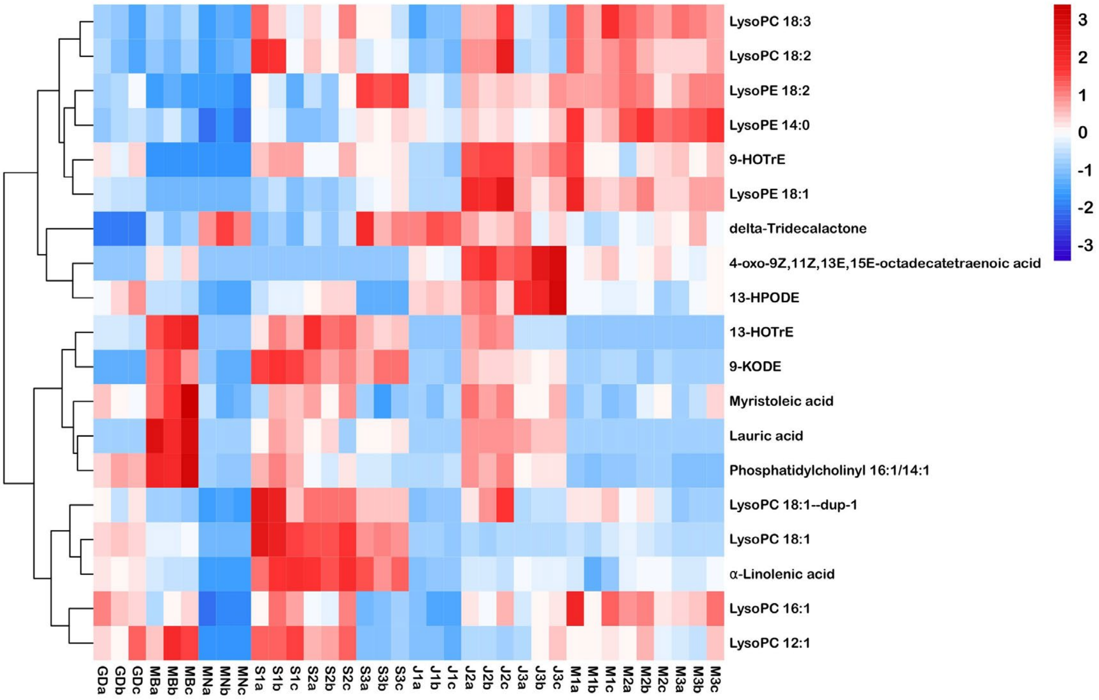

Figure S9. Heat map analysis of differential lipids. GD, Guangdong, China; MB, Northern Fujian, China; MN, Southern Fujian, China; MM, Myanmar; LK, Sri Lanka; JP, Japan. Red indicates high content and green indicates low content. $P < 0.05$.

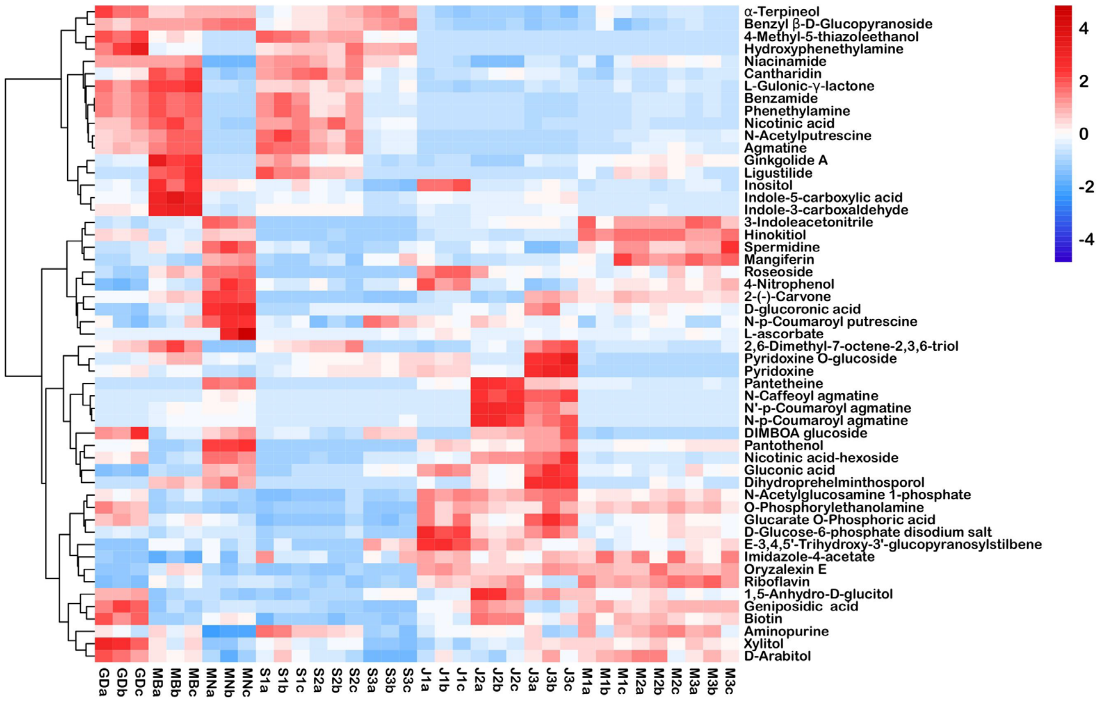

Figure S10. Heat map analysis of differential others. GD, Guangdong, China; MB, Northern Fujian, China; MN, Southern Fujian, China; MM, Myanmar; LK, Sri Lanka; JP, Japan. Red indicates high content and green indicates low content. $P < 0.05$.

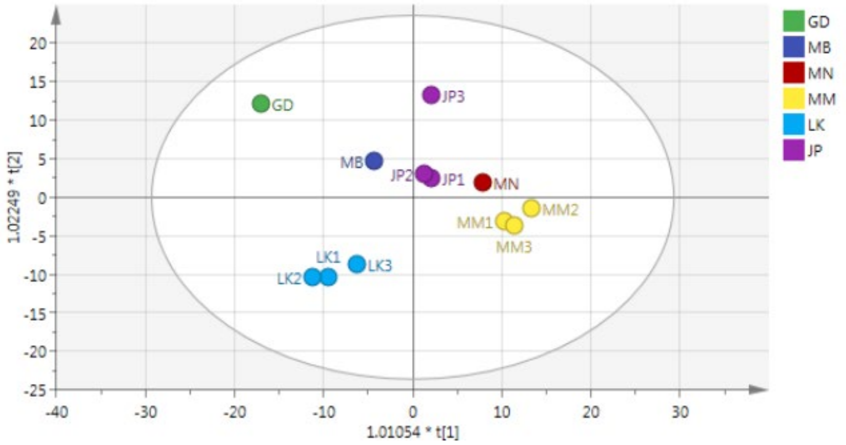

Figure S11. O2PLS score chart. GD, Guangdong, China; MB, Northern Fujian, China; MN, Southern Fujian, China; MM, Myanmar; LK, Sri Lanka; JP, Japan. $P < 0.05$.

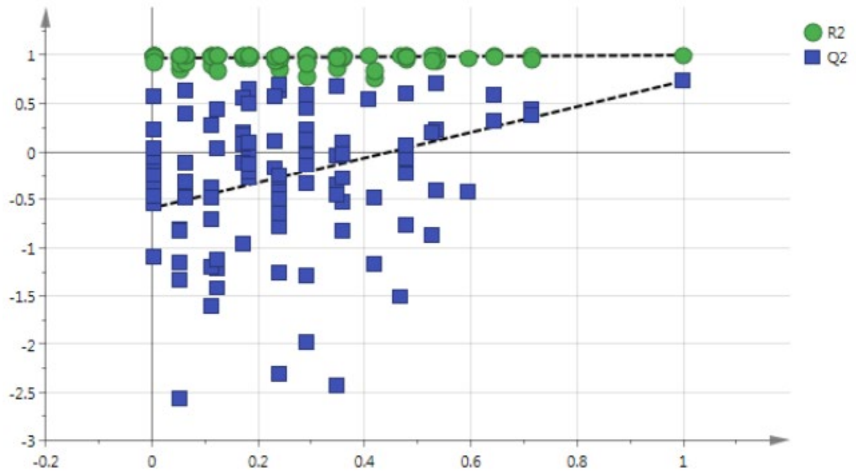

Figure S12. O2PLS permutations plot. $R^2X = 0.964$, $Q^2 = -0.589$.
